# Limited Evidence of Benefits from Clinical Trials of Human-Identical Milk Oligosaccharides for Infants

**DOI:** 10.1016/j.advnut.2026.100593

**Published:** 2026-01-14

**Authors:** Rupak Shivakoti, Barbara Laughton, Jenna Mandell, Ana Barrios-Tascon, Reshma Rajendran, Richard Glashoff, Lars Bode, Grace Aldrovandi, Louise Kuhn

**Affiliations:** 1 Department of Epidemiology, Mailman School of Public Health, Columbia University Irving Medical Center, New York, NY, United States; 2 Department of Paediatrics and Child Health, Stellenbosch University, Stellenbosch, South Africa; 3 Gertrude H Sergievsky Center, Vagelos College of Physicians and Surgeons, Columbia University Irving Medical Center, New York, NY, United States; 4 Division of Medical Microbiology and Immunology, Department of Pathology, National Health Laboratory Service, Stellenbosch University, Cape Town, South Africa; 5 Department of Pediatrics, Larsson-Rosenquist Foundation Mother-Milk-Infant Center of Research Excellence Human Milk Institute, University of California San Diego, La Jolla, CA, United States; 6 Department of Pediatrics, University of California, Los Angeles, CA, United States

**Keywords:** breastfeeding, breastmilk, prebiotics, microbiota, microbiome, infection, randomized trials, children, human milk oligosaccharides, HMO, human-identical milk oligosaccharides, HiMOs

## Abstract

Human milk oligosaccharides (HMOs) are complex carbohydrates unique to human milk, and a wealth of observational and mechanistic studies indicate that HMOs are key to infant health by supporting gut microbiota and immune development. This review synthesizes evidence from randomized clinical trials evaluating whether supplementation with human-identical milk oligosaccharides (HiMOs), i.e., synthetic HMOs, in infants and young children improves health outcomes. We identified 12 randomized clinical trials: 8 in healthy infants, 3 in special populations of infants, and 1 in young children. We selected only trials with a randomized, parallel group design; most of the included trials also had an observational human milk-fed control group. The most widely evaluated HiMO was 2′-fucosyllactose used alone or in combination with other HiMOs. In some trials, other bioactive components were included in the control and/or intervention formula groups, complicating interpretation. All trials in healthy infants confirmed the noninferiority of HiMO-supplemented formula on growth and tolerability relative to control formula. Results were mixed with respect to reductions in morbidity, and all studies were underpowered for more severe morbidity outcomes. Stool microbiota and biomarkers of inflammation and gut function generally shifted in a direction closer to human milk-fed infants with HiMO intervention. Some growth improvements were noted in association with HiMO intervention in preterm infants and in infants with severe acute malnutrition. HiMO supplementation may be a promising intervention to improve child health, but due to the heterogeneity and limitations of the clinical trials that have been undertaken, many questions remain about the nature of the benefits and the specific populations who might benefit.

**Statements of significance**

This review suggests that the evidence for clinical benefits of human-identical milk oligosaccharides (HiMOs) is limited in clinical trials of infants and young children. Although HiMOs might be a promising intervention to improve child health, current studies are heterogeneous and have key limitations that need to be addressed in future studies to better understand the nature of the potential benefits and the specific populations who may benefit.

## Introduction

Infant nutrition in the first year of life is a key determinant of health outcomes during infancy and beyond [1]. Exclusive breastfeeding is recommended as the preferred nutritional source for infants during the first 6 mo of life [2]. This is due to evidence that exclusive breastfeeding during this period is linked to age-appropriate growth, reduced infections, and reduced risk of future chronic diseases [2]. Further, breastfeeding is also associated with age-appropriate development of gut function and immunity [3].

Due to the importance of breastfeeding in health and nutrition, there is substantial interest in studying the properties of human milk and focusing on specific components that have health-promoting effects [[3], [4], [5]]. These components of human milk could be useful as infant formula ingredients, or alternatively could also be used as a supplement to human milk for mothers who may have a distinct milk profile (e.g., mothers of stunted infants [6] or mothers with HIV [7,8]). There has been strong interest recently in the role of human milk oligosaccharides (HMOs) in infant health and development [[9], [10], [11], [12]].

Following lactose and lipids, HMOs are present in human milk as the third largest component [4] and are produced directly by the lactating mother’s mammary glands [13]. Some of the common HMOs studied include neutral fucosylated HMOs such as 2′-fucosyllactose (2′-FL), neutral nonfucosylated HMOs such as lacto-N-neotetraose (LNnT), and acidic, sialylated HMOs such as 3′-sialyllactose (3′-SL) [14,15]. HMOs are not digested by the infant gastrointestinal (GI) tract and are selectively metabolized by health-promoting bacteria such as *Bifidobacterium infantis* (*B. infantis*) [[9], [10], [11]] as well as many other bacterial species, e.g., *Bacteroides* [16,17]. Some of these bacteria then generate short-chain fatty acids and many other metabolites [18,19] that are important for the development of infant gut function, immunity, and microbiome [4,[18], [19], [20]]. In addition, HMOs have antimicrobial properties or modulate epithelial and immune cell responses independent of microbes [12].

A wealth of observational studies and mechanistic studies indicate that HMOs are key to infant health by supporting gut microbiota and immune development [15,21]. Many infant formula formulations now include other non-HMO oligosaccharides such as galactooligosaccharides (GOS) and fructooligosaccharides (FOS), in part to attempt to mimic the prebiotic effects of HMOs present in human milk [22,23]. Recently, human-identical milk oligosaccharides (HiMO) [24], i.e., synthetic HMOs, have also been manufactured and added to infant formula. However, if HiMOs are to be used as interventions, clinical trials are needed. Here, we critically evaluate and synthesize the evidence from clinical trials evaluating whether HiMOs given as interventions to infants and young children improve health outcomes.

## Methods

### Search strategy

**FIGURE 1:**
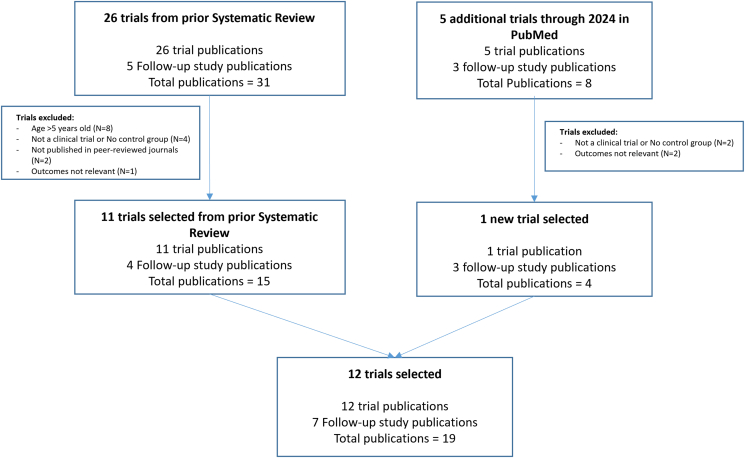
Selection of studies. Flowchart showing the initial selection of articles, the criteria used to exclude articles, and the final selection of articles for this review.

For this review, we focused on studies of randomized clinical trials of children aged <5 y published in English. There were no restrictions on studies by gestational age at birth or clinical condition. Our eligibility criteria included studies with a randomized trial design, with a parallel control group, focused on longer interventions (compared with 1-time acute challenge), studying outcomes of interest (e.g., growth, morbidity such as infections, and markers of inflammation and gut function), and a full article published in a peer-reviewed journal. Our strategy to identify relevant articles was first to critically review the articles published through 2022 in a systematic review of this topic [25], and then to search PubMed using search terms detailed in Supplementary Table 1 to identify any studies published after the review or missed by the review (Figure 1). The systematic review included publications from 26 clinical trial studies and 5 follow-up publications on these studies [25]. We excluded 8 of the clinical trials in the systematic review as they were on older children (*n* = 1; 6‒12 y old) or adults (*n* = 7). We also excluded 5 trials from the prior review because of major design flaws or were not relevant to the outcome of interest including 2 studies with no control groups [26,27, 1[26,27], 1 was an open-label prospective study [28, 1[28], 1 was a 1-time food challenge study [29], whereas another was excluded for focusing only on outcomes of tolerance [30]. Other studies from the prior review were excluded for not being published in peer-reviewed journals [31] and lacking key details of study design and conduct (e.g., only abstract published [32]). In our new review of papers published after the prior review, we identified 5 more potential studies, with only 1 of these studies meeting our quality criteria. The reasons for exclusion in the new review were 1 study with no control group [33], another with an open-label prospective study design [34], and 2 studies as 1-time challenge studies to assess iron absorption [35,36]. After our review, we identified 12 primary studies (11 from prior systematic review and 1 from a new review) meeting the above criteria reported in 19 publications [[37], [38], [39], [40], [41], [42], [43], [44], [45], [46], [47], [48], [49], [50], [51], [52], [53], [54], [55]].

### Data extraction and qualitative synthesis

For each study that met our criteria, we extracted information and conducted qualitative analysis on study population characteristics, geographic location, intervention details (HiMO type, dose, duration, method of administration, and control group), and outcomes. We then conducted a critical analysis of the study design and quality of studies by focusing on whether the study was randomized, double-blind, and whether they further had any other comparison groups. We also assessed other aspects of study design and quality, such as sample size, retention, adherence, and the type and methods of outcome assessment. We focused on clinical and methodological heterogeneity between studies based on the above characteristics. Finally, we summarized the direction of the outcomes (no effect or positive/negative effects) with the intervention for each of the included studies. The primary outcomes were related to growth (anthropometrics related to weight, length, and head circumference measures), infections, gut function (gut integrity/maturation and tolerance), and inflammation.

## Results

### Description of the included studies

We identified 12 randomized clinical trials reported in 19 publications: 8 trials in healthy infants, 3 trials in special populations of infants, and 1 trial in young healthy children (Table 1). All the studies of healthy infants were conducted in Europe and the Americas, 2 of the 3 studies in infants with special conditions were conducted in Asia, the other in Europe. The 1 trial of young children was conducted in China. Special populations included infants with cow’s milk protein allergy (CMPA) [50] or with severe acute malnutrition (SAM) [53], and 1 among preterm infants [52]. All the studies were funded by industry sponsors, except for the study of infants with SAM [53], which was funded by a non-profit organization.

**TABLE 1:**
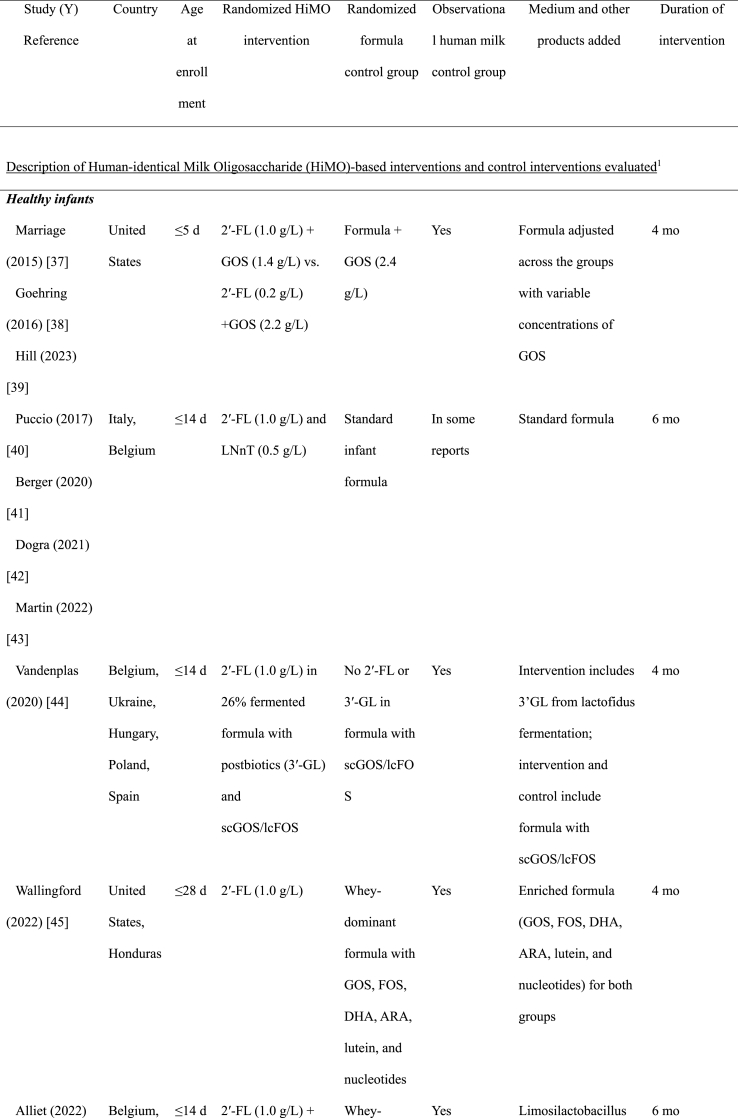

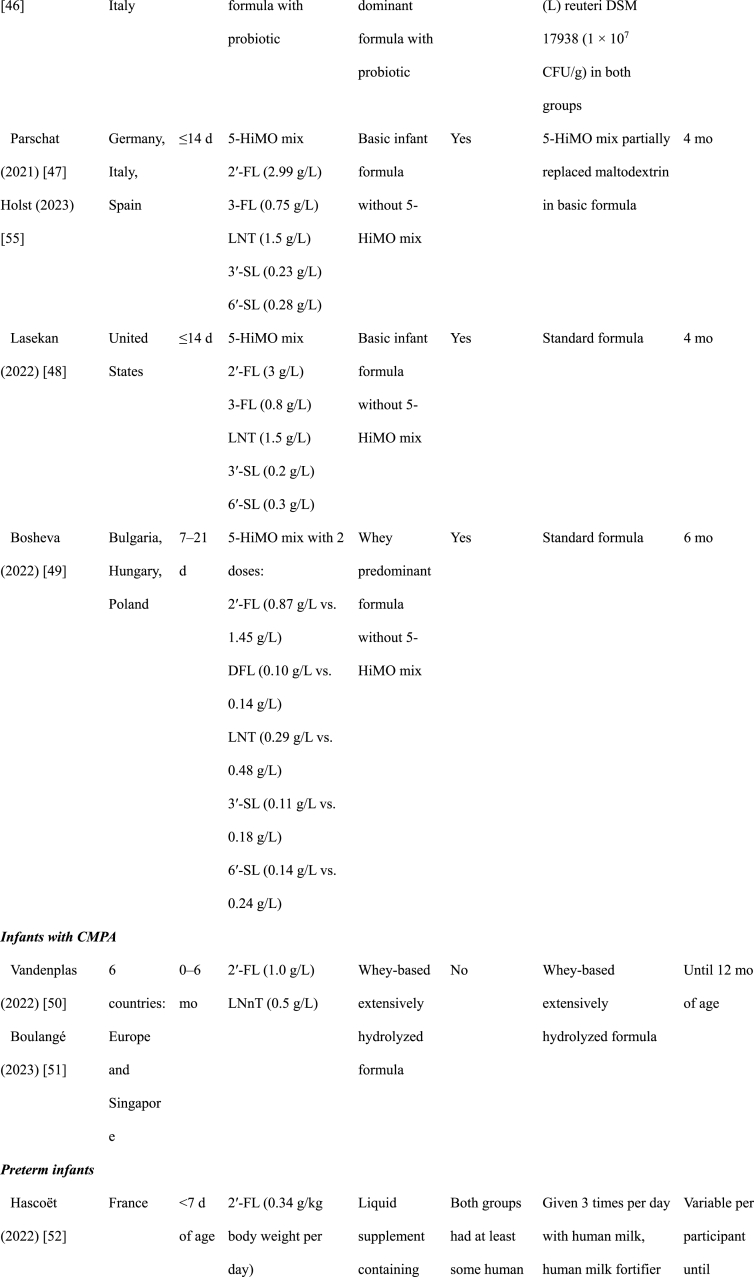

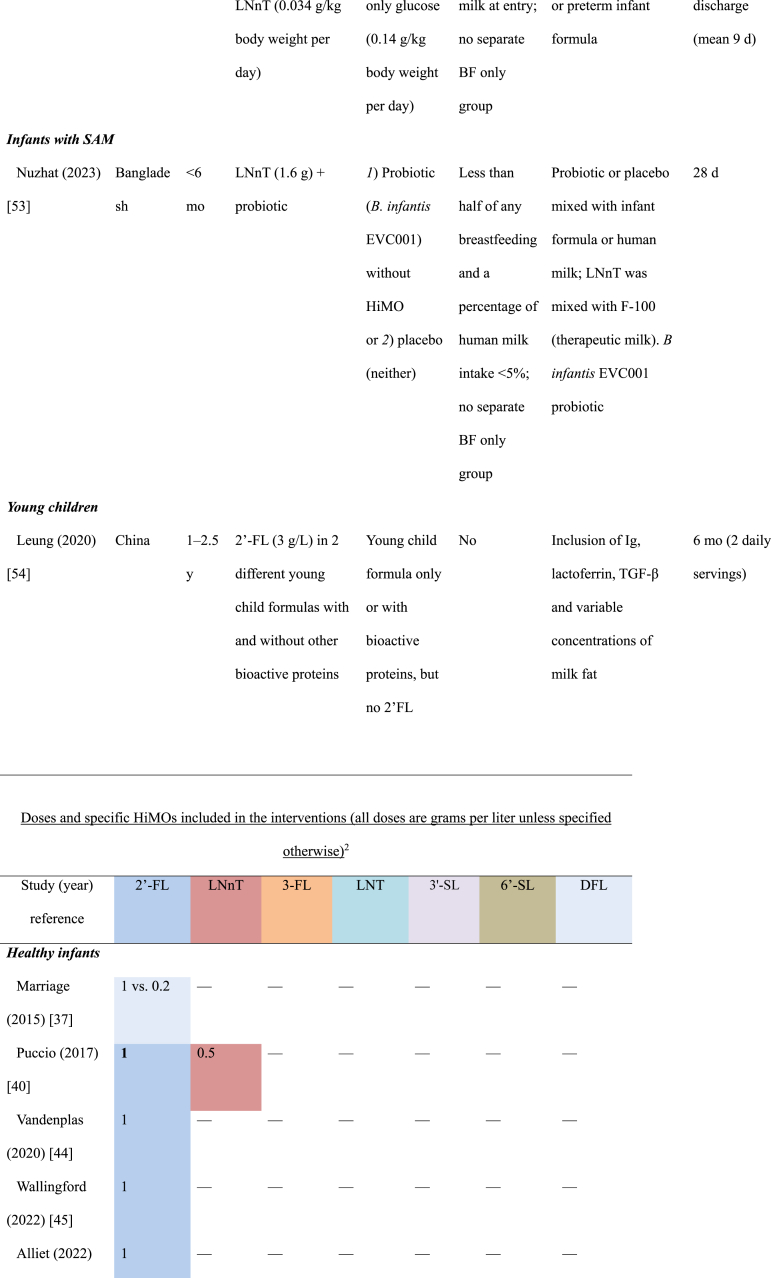

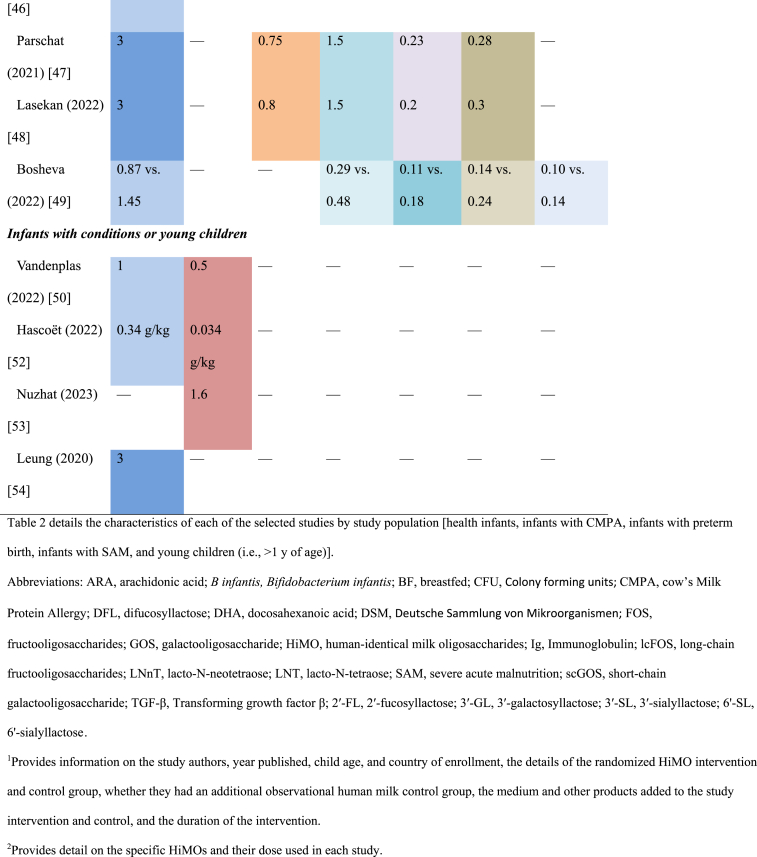
Study characteristics

### Quality of included studies

Only trials that met the minimum design criteria detailed in the methods were included in the review. All the included studies had a randomized design, and in all but 2 exceptions the randomized design was to parallel groups of different formula (Table 2) [[37], [38], [39], [40], [41], [42], [43], [44], [45], [46], [47], [48], [49], [50], [51], [52], [53], [54]]. The exceptions were the trials in preterm infants and infants with SAM. Here, infants were randomized, maintaining their background feeding practices, to either a HiMO intervention or to a placebo/active control. Most of these trials also included an observational (i.e., not randomized) control group of breastfeeding infants. This is a helpful comparator group to investigate how the intervention groups with and without HiMO compare with healthy breastfed infants. Further, all but the study in infants with SAM was also double-blind. The study of infants with SAM was single-blind, with only the caregivers blinded, but not the study staff assessing outcomes (Table 2).

**TABLE 2:** Study design and quality of included studies

Study	Randomized parallel formula group	Double-blind (formula groups)	Observational human milk control group	*n* in HiMO Intervention group(s)	*n* in no HiMO intervention and/or control group(s)	*n* in BF group	Retention rate, %	Adherence with intervention
Healthy infants								
Marriage (2015) [37] Goehring (2016) [38] Hill (2023) [39]	Y	Y	Y	104 and 109	101	106	∼80	Intake and 2’FL uptake in plasma are reported
Puccio (2017) [40] Berger (2020) [41] Dogra (2021) [42] Martin (2022) [43]	Y	Y	Included in some reports	88	87	38	∼70	Intake reported
Vandenplas (2020) [44]	Y	Y	Y	108	107	61	∼82	90‒98%; intake reported
Wallingford (2022) [45]	Y	Y	Y	66	66	89	∼80	100% compliance via diary reports, but not defined
Alliet (2022) [46]	Y	Y	Y	144	145	60	∼70	∼90% based on >80% compliant on feeding regimen
Parschat (2021) [47] Holst (2023) [55]	Y	Y	Y	113	112	116	∼79	>97%
Lasekan (2022) [48]	Y	Y	Y	130	129	104	<70	Intake reported
Bosheva (2022) [49]	Y	Y	Y	153 and 158	155	69	∼75	Not reported
Infants with conditions								
Vandenplas (2022) [50] Boulangé (2023) [51]	Y	Y	N	97	97	—	∼70	Not reported
Hascoët (2022) [52]	Y HM included	Y	Included in randomized	43	43	—	∼83	Intake of each feeding type reported
Nuzhat (2023) [53]	Y HM included	Single-blind	Included in randomized	23	23 (probiotic alone) and 21 (placebo)	—	Not reported	Not reported
Young children								
Leung (2020) [54]	Y	Y	N	114 and 114	114 and 114	—	>95	∼85%

Table 2 shows the details related to the study design and conduct that impact the quality of the included studies. This includes whether the study had a randomized parallel design for the intervention and control groups, whether it was double-blinded, if the study had an observational human milk control group, the number of participants in each of the study groups, the retention rate, and information on the adherence to the intervention.

Abbreviations: BF, breastfed; HiMO, human-identical milk oligosaccharides; HM, human milk; *n*, number of participants; N, no; Y, yes; 2′-FL, 2′ fucosyllactose.

We also assessed other features of clinical trials that impact quality and risk of bias, including the sample size, retention, adherence/compliance, and rigorous outcome assessment. The sample size in studies of healthy children ranged from 66 per group to 158 per group in the randomized arms, with most of the studies having >100 participants per group (Table 2). However, the sample size in studies of infants with preterm birth (43 per group) and SAM (23 per group) was much smaller, and likely underpowered for primary and secondary outcomes (Table 2).

The retention of the enrolled participants was variable. The study of healthy young children had >95% retention, but the rest of the studies for healthy infants and infants with clinical conditions had retention of around ≤80% (Table 2). Although a range of reasons for the lower levels of retention were presented, such as the COVID pandemic [48], the most common reasons for attrition were parent withdrawal or loss-to-follow-up without further details provided. There was no evidence of differential attrition across the groups. The retention rate was not reported for children with SAM [52,53] (Table 2).

Related to adherence/compliance to the assigned study product, key details were lacking in multiple studies related to whether adherence was measured (Table 2), the methods of assessment (including whether objective measures were obtained), definitions and cutoffs, adherence rates, and analytical approach for those with low adherence. In studies that measured adherence (Table 2), the adherence levels were generally similar between the intervention and placebo groups, although the methods of assessment were variable, including through concentrations of HiMO in plasma samples, mean daily amount of formula consumed (e.g., based on weight or volume of returned product), feeding diaries, and questionnaires given to caregivers.

The outcomes assessed by all the studies for growth and GI symptoms were rigorous, with longitudinal and frequent follow-up. The other outcomes, such as morbidity/ adverse events (AEs), gut microbiome and inflammation were also rigorous although with limitations related to their data collection (e.g., parent-reported morbidity/AEs [40,54], and either limited coverage (e.g., only 16S rRNA sequencing for microbiome or limited panel of metabolites or inflammation markers) or time-points for marker assessments [38,41,45,46,49,51,54]).

### Description of the HiMO interventions

The oligosaccharide 2′-FL was the most frequently used HiMO component and was used alone or in combination in all trials among healthy infants (Table 1) [[37], [38], [39], [40], [41], [42], [43], [44], [45], [46], [47], [48], [49], [50], [51], [52], [53], [54]]. All of the 8 trials in healthy infants included 2′-FL used at dosages ranging from 0.87 g/L to 3 g/L, with 1 trial including an additional arm with a very low concentration (0.2 g/L). Four of the trials in healthy infants used 2′-FL without other HiMOs, 1 trial combined 2′-FL with LNnT, and 3 trials evaluated mixtures of 5 HiMOs [2′-FL, 3-FL, lacto-N-tetraose (LNT), 3′-SL, 6′-SL in 2 trials, and 2′-FL, difucosyllactose, LNT, 3′-SL, 6′-SL in the other trial]. In special populations, 1 trial used only LNnT and 2 had 2′-FL combined with LNnT. The 1 trial of young children used 2′-FL alone (Table 1).

In the 8 healthy infant trials, 4 of the trials included additional bioactive components in both intervention and control groups, including variable concentrations of GOS in 1 trial, short-chain GOS/long-chain FOS in another, GOS, FOS, Docosahexaenoic acid (DHA), Arachidonic acid (ARA), lutein, and nucleotides in a third trial, and the probiotic *Limosilactobacillus (L) reuteri* in the other trial. The trial that added short-chain GOS/long-chain FOS to both intervention and placebo groups also had an additional bioactive component–fermented formula with postbiotics (i.e., inanimate microorganisms or their components that can have a health benefit to the host [56]) 3′-galactosyllactose- in the intervention arm only. In the trial of infants with SAM, the active arms included *B. infantis* probiotics, a bacterium that can ferment HiMOs [9‒11], either with or without the HiMO, compared with a control formula with neither. Only the study of infants with preterm birth used HiMOs mixed with human milk, whereas the other studies used HiMOs mixed with formula (Table 1). The study of infants with SAM included breastfeeding infants, but the HiMO was mixed with F-100 formula, although the probiotics and placebo could be mixed with breast milk [53].

Related to intervention duration, in the studies of healthy children, the intervention was between 4 and 6 mo in length (Table 1). Except for the study from China, which gave the intervention to children 1 to 2.5 y of age for a 6 mo duration [54], the other studies of healthy children initiated the intervention during the first month of life, thus covering the critical period where HiMO could have the most impact. The study of children with CMPA enrolled infants 0 to 6 mo of age and provided the intervention through 12 mo [50]. The wide range for age of enrollment was also present in the SAM study, with this study only providing 1 mo of intervention [53]. The study of preterm birth only provided the intervention until discharge from the neonatal unit (variable per infant) [52]; although this is also likely too short for sustained effects, it may still have short-term effects that are crucial for successful discharge from neonatal units (Table 1).

### Effects of the interventions on infant outcomes

#### Growth

Seven of the 8 studies in healthy infants reported on growth parameters as a primary outcome. In all of these studies, the HiMO intervention had noninferior anthropometric parameters, including gain in weight, length, and head circumference, and attained age-adjusted *z*-scores compared with the control formula without HiMOs. Six of these studies also had breastfed infants without intervention as an additional comparison group. Growth was largely similar and within the normal range in breastfed infants, with generally lower age-adjusted *z*-scores relative to the formula groups, consistent with expectations. In studies of infants with CMPA and in the study in young children, anthropometric parameters were noninferior in the HiMO group compared with the control formula (Table 3) [[37], [38], [39], [40], [41], [42], [43], [44], [45], [46], [47], [48], [49], [50], [51], [52], [53], [54]].

**TABLE 3:** Outcomes investigated and findings

Study (y) reference	Growth	Tolerability	Results for growth and tolerability		Morbidity	Results for morbidity		Markers of mechanism of action	Results for markers
			Relative to FF	Relative to BF		Relative to FF	Relative to BF		
**Healthy infants**									
Marriage (2015) [37] Goehring (2016) [38] Hill (2023) [39]	Weight, length, and head circumference gains per day	GI symptoms and tolerability	Noninferior growth; tolerance similar	All groups are quite similar for growth; some tolerability indicators are better with BF∗	AEs	No difference in overall AE/SAEs; “Infection and infestation” AEs were less in 1 formula group (0.2 g/L 2′-FL)^1^	Not reported	Inflammatory cytokine profiles Immune cell profiling Microbial metabolites	Less inflammation with 2′-FL shifts in bile acids
Puccio (2017) [40] Berger (2020) [41] Dogra (2021) [42] Martin (2022) [43]	Weight, length, and head circumference gains per day	GI symptoms and tolerability	Noninferior growth; some improvements in tolerability indicators^1^	—	AEs; infections	Reduced reports of bronchitis, LRTI, antipyretics, and antibiotics^1^	BF group not included in primary report	Stool microbiota (16S rRNA and shotgun metagenomic sequencing) Stool metabolites Gut health markers	Shifts toward BF and find an association with morbidity
Vandenplas (2020) [44]	Weight, length, and head circumference gains per day	GI symptoms and tolerability	Noninferior growth; tolerance similar	A bit lower weight gain in BF^1^; differences in some tolerability indicators	AEs	No difference with the control formula	A bit worse than BF	Not studied	—
Wallingford (2022) [45]	Weight gain per day	Not reported	Noninferior growth	Similar growth to BF	AEs	No difference	Incidence of GI disorders is worse than that of BF	Stool microbiota (metagenomic species approach) Genes for 2′-FL metabolism	No difference in taxonomy or diversity; some shifts noted, but not comparable to BF
Alliet (2022) [46]	Weight, length, and head circumference gains per day	GI symptoms and tolerability	Noninferior growth; tolerance similar	Growth comparable, but BF WAZ a bit lower; some differences in tolerability (higher stool frequency, flatulence less in BF)^1^	AEs	Comparable between the 2 groups	Similar to the formula groups	Stool microbiota (16S rRNA sequencing) Fecal pH, organic acids, biomarkers of gut health	Shifts toward microbiota and other biomarkers are more similar to BF
Parschat (2021) [47] Holst (2023) [55]	Mean weight over time	GI symptoms and tolerability	Noninferior growth; some improvements in tolerability indicators^1^	Lower weight for BF and differences in length^1^; slight indication HiMO group tolerance closer to BF^1^	AEs and health care utilization	AEs similar; less health care utilization than the control formula^1^	AEs are mostly similar to BF; the lowest HC utilization in BF^1^	Stool Microbiome (composition and function)	Shifts towards microbiome profile more similar to BF
Lasekan (2022) [48]	Weight, length, and head circumference gain per day	GI symptoms and tolerability	Noninferior growth; some improvements in tolerability indicators^1^	Growth comparable; slight indication HiMO group tolerance closer to BF^1^	AEs	AEs similar; benefit of reducing HCP visits in formula group^1^	Both formula groups have slightly higher AEs than BF	Not studied	—
Bosheva (2022) [49]	Not reported	Not reported	—	—	Not reported	—	—	Stool microbiota (shotgun metagenomics) Fecal pH, organic acids, biomarkers of gut health	Improved gut maturation; enrichment of *Bifidobacteria*, lower toxigenic *Clostridiorides,* closer to BF infants
**Infants with conditions**									
Vandenplas (2022) [50] Boulangé (2023) [51]	- Weight, length, and head circumference gain per day	GI symptoms and tolerability	Daily weight gain and growth are noninferior; tolerance is similar	—	CMPA symptom resolution; AEs and morbidity, including infections	No difference in CMPA symptoms or AEs; Significantly lower URTIs and ear infections^1^; trend to lower GI and LRTI	—	Stool microbiota (shotgun metagenomics)	Enrichment of *Bifidobacteria* and delayed shift to adult-like patterns (correction of dysbiosis typical of CMPA)
Hascoët (2022) [52]	Weight, length, and head circumference gain per day and attained *z*-scores	GI tolerability Time to full enteral feeds	Length and head circumference *z*-scores better are in the HiMO group^1^; Noninferiority for time to enteral feeds	—	AEs	Similar across groups	—	Not investigated	—
Nuzhat (2023) [53]	Weight and length gain per day for 4 wk after the supplementation period	Not reported	Weight gain in HiMO + probiotic was greater than placebo but less than probiotic alone^1^	—	Not investigated	—	—	Not investigated	—
**Young children**									
Leung (2020) [54]	Weight and Height *z*-score	GI symptoms and tolerability	Growth comparable; Some of the intervention groups had fewer days with hard stool^1^	—	AEs; respiratory and GI infections incidence and duration	AEs similar; unexpected increase in duration of URTI for 1 of the intervention groups^1^	—	Stool microbiota (16S rRNA sequencing)	No differences

Table 3 specifies the outcomes investigated by each study and provides a summary of the results. The outcomes include growth, tolerability, morbidity (including AEs), and markers of mechanism of action (e.g., inflammation, microbiome, and metabolites), and results are shown comparing the intervention to placebo, and the study groups to BF control groups (when available). For results related to growth, tolerability (at least some of the indicators), and morbidity. The significance results are not shown for markers of mechanisms of action due to the different types and number of markers assessed.

Abbreviations: AE, adverse event; BF, breastfed; CMPA, cow’s milk protein allergy; FF, formula fed; GI, gastrointestinal; HC, healthcare; HCP, healthcare provider; HiMO, human-identical milk oligosaccharides; LRTI, lower respiratory tract infection; rRNA, ribosomal ribonucleic acid; SAE, serious adverse event; URTI, upper respiratory tract infection; WAZ, weight-for-age *z*-scores; 2′-FL, 2′-fucosyllactose; pH.

1 Significant (i.e., *P* values < 0.05) differences noted by the investigators.

In the 2 trials that focused on infants at risk for poor growth, some improvements in growth outcomes were observed. In a study in France, 86 preterm infants (27‒33 gestational age and <1700 g birthweight) in neonatal units were randomized to receive either a HiMO supplement (0.34 g/kg 2′-FL and 0.034 g/kg LNnT) or a placebo containing only glucose from birth-7 d through discharge [52]. Similar weight gain and weight-for-age *z*-scores through discharge were noted for both groups, and time to full enteral feeds was similar. However, the HiMO supplement group had significantly higher length-for-age (LAZ) and head circumference-for-age (HCAZ) Z scores compared with placebo (Table 3).

A trial in Bangladesh focused on 67 infants with SAM ∼6 mo of age. These infants were receiving nutritional rehabilitation for SAM and were then randomized to either a probiotic (*B. infantis*), a synbiotic (*B. infantis* + 1.6 g/L of HiMO LNnT), or a placebo [53] for 1 mo. Both infants in the HiMO-based synbiotic arm and the probiotic alone arms gained significantly more weight than the placebo arm, although weight gain was highest in the probiotic alone arm [53] (Table 3).

#### Tolerability and GI symptoms

Six of the 8 trials in healthy infants, the trial in infants with CMPA, the trial in preterm infants, and the trial in young children assessed GI tolerability using various scales and questionnaires [37,40,44,[46], [47], [48],^50^,^52^,^54^]. All studies found HiMO interventions to be tolerable and safe in both healthy infants and in infants with clinical conditions (Table 3). Overall, in healthy infants and the study involving infants with CMPA and preterm birth, symptoms such as vomiting/spitting-up, stool consistency, softness and frequency, flatulence, and regurgitation were similar between the intervention group and comparison groups [37,40,44,[46], [47], [48],^50^,^52^,^54^]. In some studies [40,47,48,54], HiMOs resulted in some of the stool characteristics/symptoms improving, including multiple studies reporting more frequent stools that were soft, and were more similar to breastfed infants as compared with the control group (Table 3).

#### AEs/morbidity

Seven of the 8 trials in healthy infants, the trial in infants with CMPA, the trial in preterm infants, and the trial in young children provided some assessment of morbidity or AEs (Table 3).

Four of the 7 trials in healthy infants found some reductions in some markers of AEs and morbidity. In a randomized trial of 175 healthy infants aged <2 wk from Belgium and Italy [40], infants randomized to a formula with 1 g/L of 2′-FL and 0.5 g/L of LNnT for 6 mo had significantly lower incidence of bronchitis and lower respiratory tract infections through 12 mo of age compared with infants receiving a control formula without HiMOs. Similarly, the infants on HiMOs were reported to have significantly less antibiotic use and also lower rates of otitis media/ear infections [40]. In another trial of healthy infants from the United States with 2 intervention groups (0.2 g/L or 1.0 g/L of 2′-FL) compared with a control formula [37], the overall rates of AEs were similar between the groups, but AEs related to infections were less in 1 of the formula groups (0.2 g/L). In 2 other trials, the intervention groups with HiMO had improved morbidity outcomes as assessed through reduced health care utilization, with rates closer to breastfed infants [47,48]. The other 3 trials in infants found no difference in AEs between the HiMO intervention and control formula groups. It should be noted that severe morbidity events, given the population of healthy infants, were rare in all trials, and markers of morbidity were generally higher in formula groups than in the breastfeeding groups.

The trial in infants with CMPA found that while the group receiving HiMOs was similar to the control group on CMPA symptom resolution and overall AEs, they had significant reductions in the frequency of upper respiratory tract infection (URTI) and ear infections, with also nonsignificant decreases in lower respiratory tract infection and GI tract infections (GITI) compared with controls without HiMOs [50]. The trial in preterm infants did not observe differences in AEs.

In the trial of 114 young children aged 1 to 2.5 y from China [54], formula-based interventions containing 3 g/L of 2′-FL given for 6 mo were largely similar compared with control in the primary outcomes of URTI incidence and duration of GITI [54], although there was an unexpected increase in the duration of URTIs in one of the HiMO intervention groups.

#### Gut maturation/function and microbiome

Six of 8 studies in healthy infants investigated biomarkers of gut maturation and function, including characterizing fecal microbiota. Most of these found some improvements associated with the HiMO interventions, included better epithelial barrier function and intestinal immunity, increased production of certain metabolites (e.g., microbial fermentation products of dicarboxylic acid and short-chain fatty acids, along with secondary bile acids), and a microbiota profile more similar to breastfed infants (e.g., similar diversity metrics, higher *B. infantis*, lower toxigenic *Clostridium dificile*) [39,[41], [42], [43],^45^,^46^,[49], [55]]. However, there was substantial variability between the studies in the markers studied, the methods used to assess gut function and the microbiome, and the results for specific outcomes. For example, although some studies noted clear changes in the microbiome with the intervention [39,[41], [42], [43],^46^,[49], [55]], 1 study [45] did not note major differences in the composition, with some differences noted in function (e.g., increases in gene-level abundance of glycosyl hydrolase).

In the study of infants with CMPA [50], supplementation with 2′-FL and LNnT was able to partially correct gut microbial dysbiosis common to infants with CMPA in comparison to the control formula group. It showed increases in *Bifidobacteria* and a delayed shift toward an adult-like pattern [51], and there were also metabolomic changes, including increased fecal amino acid degradation and bile acid conjugation with the intervention [51]. These changes could explain the observed reduced GITI with the intervention.

The study of young infants in China found no differences between the intervention and placebo in the gut microbiota profile [54]. The 2 trials with infants with preterm birth and SAM did not assess markers of gut function or the gut microbiome.

#### Inflammation

Two studies [38,49] of healthy infants assessed the effect of HiMOs on inflammation. In a United States study, 311 infants ≤5 d of age were randomized to 2 formula-based intervention groups with different concentrations (1.0 g/L and 0.2 g/L) of 2′-FL or a control formula group for 4 mo. They also had a breastfed infant group without any intervention for comparison purposes [37,38]. They observed that the 2′-FL intervention groups, similar to the breastfed infants, had lower concentrations of plasma inflammatory cytokines, including IL-receptor antagonist (IL-1ra), IL1α, IL1β, and TNFα [38]. They also observed reduced concentrations of cytokines in ex vivo respiratory syncytial virus-stimulated peripheral blood mononuclear cultures [38].

In another study in Europe, 466 infants 7 to 21 d of age were randomized to either 2 intervention groups receiving formula for 6 mo with different concentrations (0.87 g/L and 1.45 g/L) of a 5-HiMO product (2′-FL, difucosyllactose, LNT, 3′-SL, and 6′-SL) as compared with a control group receiving formula without HiMOs [49]. Both the intervention groups improved gut barrier function and supported the development of the intestinal immunity, as indicated by higher fecal secretory Ig A, lower α-1 antitrypsin, and lower calprotectin concentrations, compared with the control group without HiMO [49]. This study also included a breastfed group without any intervention and observed that the HiMO-based groups were more similar in profile to the breastfed infants [49].

## Discussion

Our review was focused on randomized clinical trials on the effects of HiMO supplementation on infant outcomes for healthy infants and those with clinical conditions. Healthy infants on formula with one or more HiMOs had noninferior growth and tolerability compared with control formula groups, whereas some improvements in growth were noted for preterm infants and infants with SAM. Although some studies reported reductions in morbidity, especially infectious morbidity, with HiMOs, the results were mixed, and studies were underpowered for severe morbidity. Biomarkers related to inflammation and gut function showed improvements and a shift toward a profile more similar to breastfed infants. However, due to the heterogeneity and limitations of these studies, it is difficult to directly compare the studies for interpretation. Further well-designed studies are needed to better understand the impact of HiMOs on health outcomes and the populations most likely to benefit from these interventions, such as children with various clinical conditions from diverse populations.

In healthy children, there is consistent evidence across multiple studies that formula with HiMO intervention has noninferior anthropometric parameters compared with controls on formula without HiMOs [37,40,44,48,54] and were in the normal range similar to breastfed infants [37,[44], [45], [46], [47], [48]]. Although there are known differences in growth parameters, for example breastfeeding is linked to more age-appropriate growth as compared with formula [2], it should be noted that the underlying populations studied were mostly healthy infants (e.g., no apparent malnutrition status such as SAM or obesity), thus the growth parameters were largely similar in breastfeeding and formula fed (with or without HiMOs). Although the noninferiority with HiMOs as it relates to growth, as well as the safety and tolerability, is reassuring, the clinical need for HiMOs in healthy children is not clear.

For CMPA infants [50], the growth patterns were similar between the intervention and placebo groups, but had minor upward increases compared with WHO references. The authors suggest that this is in part explained by the known increase in growth acceleration in infants fed extensively hydrolyzed formula compared with breastfed infants (used for WHO references), and could reflect potential catch-up growth from CMPA when they initiate extensively hydrolyzed formula [50]. Of note, the improvement in growth is consistent with other studies of CMPA that we excluded from this review due to eligibility criteria [26,27,33]. In contrast, children with SAM [53] or preterm birth [52] showed improvements in certain growth parameters with HiMO supplementation. However, these studies are small and seem to impact some but not other anthropometric outcomes. Further, the study of infants with SAM only gave the intervention for one month, which may not be sufficient to observe effects, and did not have a HiMO alone group, and the probiotic alone arm had better outcomes than the synbiotic arm with HiMOs. Thus, further larger well-designed studies in these populations with growth deficits such as SAM and preterm birth are needed.

HMOs are hypothesized to affect infant growth through various mechanisms that result in catch-up growth in undernourished populations. For example, HMOs can impact the development of the microbiome and infant immune system [19,52,57], which are key factors in infant growth. In line with this, a study in gnotobiotic mice showed that bovine milk oligosaccharides were able to rescue growth impairments that resulted from fecal transplant from infants with stunting [6]. However, many bovine milk oligosaccharides are structurally different from HMOs. Further, HMOs may affect gut function, including by microbes producing short-chain fatty acids during HMO digestion, and ultimately impact infant growth [19,58]. Our review suggests that further studies are needed to test whether HiMO supplementation has a role in improving age-appropriate growth in settings of undernutrition. It is also not clear, and further study is needed on whether and how HiMO supplementation are effective intervention to improve age-appropriate growth in other populations, such as populations with overnutrition (e.g., obesity, where dietary fiber is thought to play a protective role) or other clinical conditions where infant growth is impacted (e.g., maternal HIV infection [10]).

Multiple studies assessed the impact of HiMO supplementation on AEs and morbidity. Several studies in healthy infants and one with CMPA observed reductions in markers of morbidity, including lower rates of infectious morbidity (especially respiratory infections), less antibiotic use, and reduced health care utilization in the HiMO group as compared with formula control. Potential mechanisms for this observed reduction of morbidity include their ability to reduce susceptibility to infections through their antimicrobial and anti-adhesive properties, and their ability to impact the gut microbiome and immunity [18,19,59].

However, other studies did not observe differences in AEs or morbidity between the intervention and control groups. The reasons for the discrepancy are not clear. One possible reason could be differences in study characteristics, for example, 1 study that did not observe reductions in infections was conducted in an older age group (1‒2.5 y), and possibly it is more effective in reducing infections at a younger age (when it would be naturally given to infants). However, larger studies powered on infectious morbidity are needed to better understand the effect of HiMOs on overall and infectious morbidity and AEs.

Of note, in the studies that observed reductions in morbidity with the intervention, the morbidity in HiMO groups was more similar to that of the breastfed infant controls. This raises interesting questions related to which comparison group, the randomized control formula group or the breastfeeding group, is most informative. Although the randomized control formula group is important (i.e., a control formula without the intervention, allowing for blinding and randomization) for direct comparisons, the parameters of interest related to morbidity, growth, and other outcomes would be those observed in the breastfeeding control group. Reassuringly, most of the studies reported here had both control formula and breastfeeding groups that allowed for direct comparisons with the intervention, although it should be noted that there are likely differences in study population characteristics by breastfeeding status. As done in the study of infants with SAM, future studies should also provide HiMOs to breastfeeding infants to test whether there are additional benefits in specific populations of interest [[6], [7], [8]].

HiMOs were safe and tolerable, with some studies even showing improvements in some GI symptoms with the intervention. HiMO supplementation also generally resulted in improved gut function and reduced gut and systemic inflammation. Further, in both healthy infants and infants with CMPA, as previously discussed, HiMO supplementation resulted in improved gut maturation and function, and reduced gut microbiota dysbiosis [39,[41], [42], [43],^45^,^46^,^49^,^51^,[52], [55]]. These results related to gut function, which impacts epithelial barrier integrity and microbial translocation, likely also explain lower levels of gut and systemic inflammation observed in healthy infants. Further, the short-chain fatty acids produced by bacteria during HiMO digestion also have anti-inflammatory properties. For infants with CMPA, prior studies have shown that they have gut dysbiosis with decreases in *Bifidobacteria*, and it is hypothesized that HiMOs help restore the dysbiosis, subsequently improving immunity and reducing infections such as rotavirus that have a direct impact on growth [27,50]. Of note, a key advantage of randomized controlled trials for secondary analyses of these biomarkers is that they create a well-defined comparator group to identify biological mechanisms and pathways through which HiMOs could impact health outcomes.

Based on our summary of the existing literature, we have several recommendations for further study that will allow for a better understanding of the clinical impact of HiMOs. A major limitation of the existing studies is the heterogeneity of the interventions used (i.e., different HiMOs and in different concentrations, and some with and others without other bioactive components, including probiotics), which makes it difficult to compare between studies. Although we were not able to observe differential effects of any specific HiMOs (e.g., almost all the interventions included 2’FL), it should be noted that, as different HiMOs are structurally and functionally distinct, further studies are needed that can provide direct evidence of the impact of specific HiMOs and their combinations for various outcomes. Related to the study population and outcomes being studied, the clinical indication for HiMO supplementation needs to be better justified. For example, focusing on populations at risk for poor growth or at risk for morbidity might be more informative than studying healthy infants. Given that multiple studies on this topic have been excluded from this review due to weaknesses in design, along with limitations in the included studies, we recommend that future studies address study design limitations. This includes a larger sample size (especially to be powered for morbidity outcomes), better measurement and analysis of adherence, improved retention, and more thorough biomarker measurement.

In conclusion, this review demonstrates the heterogeneity and limitations of the clinical trials conducted and suggests that while HiMOs could be promising interventions to improve child health, further trials are needed before we can reach more informed conclusions about the nature of the benefits and the specific populations who may benefit. Expanding the investigation through well-designed and conducted studies in larger, more diverse populations with clinical need is required to confirm and expand on our understanding of the potential effectiveness of specific HiMOs and their combination to improve child health.

## Author contributions

The authors’ responsibilities were as follows – RS, LK: designed research, wrote the manuscript, analyzed data and have primary responsibility for final content; AB-T, RR: contributed to data analysis (including selection of articles) and manuscript writing and review; BL, JM, RG, LB, GA: contributed to manuscript review and writing; and all authors: read and approved the final manuscript.

## Data availability

The data described in the manuscript are all available in the tables presented as well as the cited publications.

## Funding

Research reported in this article was supported by the Eunice Kennedy Shriver National Institute of Child Health and Human Development of the NIH under award number R01HD105492 to RS. The content is solely the responsibility of the authors and does not necessarily represent the official views of NIH. LB is the University of California San Diego Chair of Collaborative Human Milk Research, endowed by a generous gift from the Family Larsson-Rosenquist Foundation in Switzerland.

## Conflict of interest

The authors report no conflicts of interest.

## References

[1] Robinson S.M. (2015). Infant nutrition and lifelong health: current perspectives and future challenges. J. Dev. Orig. Health Dis..

[2] Meek J.Y., Noble L. (2022). Policy Statement: Breastfeeding and the use of human milk. Pediatrics..

[3] Carr L.E., Virmani M.D., Rosa F., Munblit D., Matazel K.S., Elolimy A.A. (2021). Role of human milk bioactives on infants’ gut and immune health. Front Immunol..

[4] Andreas N.J., Kampmann B., Mehring Le-Doare K. (2015). Human breast milk: a review on its composition and bioactivity. Early Hum. Dev..

[5] Ballard O., Morrow A.L. (2013). Human milk composition: nutrients and bioactive factors. Pediatr. Clin. North Am..

[6] Charbonneau M.R., O’Donnell D., Blanton L.V., Totten S.M., Davis J.C., Barratt M.J. (2016). Sialylated milk oligosaccharides promote microbiota-dependent growth in models of infant undernutrition. Cell.

[7] Bode L., Kuhn L., Kim H.Y., Hsiao L., Nissan C., Sinkala M. (2012). Human milk oligosaccharide concentration and risk of postnatal transmission of HIV through breastfeeding. Am. J. Clin. Nutr..

[8] Shivakoti R., Slogrove A.L., Laughton B., Shafiq M., Schoeman E., Glashoff R.H. (2022). Mitigating Infectious morbidity and Growth deficits in HIV-exposed uninfected infanTs with human Milk oligosaccharide (MIGH-T MO): a randomised trial protocol. BMJ Open.

[9] Kunz C., Rudloff S., Baier W., Klein N., Strobel S. (2000). Oligosaccharides in human milk: structural, functional, and metabolic aspects. Annu. Rev. Nutr..

[10] Li W., Wang J., Lin Y., Li Y., Ren F., Guo H. (2021). How far is it from infant formula to human milk? A look at the human milk oligosaccharides. Trends Food Sci. Technol..

[11] Bode L. (2015). The functional biology of human milk oligosaccharides. Early Hum. Dev..

[12] Bode L. (2012). Human milk oligosaccharides: every baby needs a sugar mama. Glycobiology..

[13] Sprenger N., Tytgat H.L., Binia A., Austin S., Singhal A. (2022). Biology of human milk oligosaccharides: from basic science to clinical evidence. J. Hum. Nutr. Diet..

[14] Dinleyici M., Barbieur J., Dinleyici E.C., Vandenplas Y. (2023). Functional effects of human milk oligosaccharides (HMOs). Gut Microbes.

[15] Moubareck C.A. (2021). Human milk microbiota and oligosaccharides: A glimpse into benefits, diversity, and correlations. Nutrients.

[16] Renwick S., Furst A., Knip M., Study Group DIABIMMUNE, Bode L., Danska J.S. (2025). Modulating the developing gut microbiota with 2′-fucosyllactose and pooled human milk oligosaccharides. Microbiome.

[17] Buzun E., Hsu C.Y., Sejane K., Oles R.E., Vasquez Ayala A., Loomis L.R. (2024). A bacterial sialidase mediates early-life colonization by a pioneering gut commensal. Cell Host Microbe.

[18] Plaza-Díaz J., Fontana L., Gil A. (2018). Human milk oligosaccharides and immune system development. Nutrients.

[19] Triantis V., Bode L., van Neerven R.J. (2018). Immunological effects of human milk oligosaccharides. Front Pediatr.

[20] Marcobal A., Sonnenburg J.L. (2012). Human milk oligosaccharide consumption by intestinal microbiota. Clin. Microbiol. Infect..

[21] Walsh C., Lane J.A., van Sinderen D., Hickey R.M. (2020). Human milk oligosaccharides: shaping the infant gut microbiota and supporting health. J. Funct. Foods..

[22] Mei Z., Yuan J., Li D. (2022). Biological activity of galacto-oligosaccharides: a review. Front Microbiol.

[23] Abrahamse-Berkeveld M., Alles M., Franke-Beckmann E., Helm K., Knecht R., Köllges R. (2016). Infant formula containing galacto-and fructo-oligosaccharides and Bifidobacterium breve M-16V supports adequate growth and tolerance in healthy infants in a randomised, controlled, double-blind, prospective, multicentre study. J. Nutr. Sci..

[24] Hojsak I., Dinleyici E.C., van den Akker C.H., Domellöf M., Haiden N., Szajewska H. (2025). Technical review by the ESPGHAN special interest group on gut microbiota and modifications on the health outcomes of infant formula supplemented with manufactured human milk oligosaccharides. J. Pediatr. Gastroenterol. Nutr..

[25] Schönknecht Y.B., Moreno Tovar M.V., Jensen S.R., Parschat K. (2023). Clinical studies on the supplementation of manufactured human milk oligosaccharides: A systematic review. Nutrients.

[26] Ramirez-Farias C., Baggs G.E., Marriage B.J. (2021). Growth, tolerance, and compliance of infants fed an extensively hydrolyzed infant formula with added 2′-FL fucosyllactose (2′-FL) human milk oligosaccharide. Nutrients.

[27] Gold M.S., Quinn P.J., Campbell D.E., Peake J., Smart J., Robinson M. (2022). Effects of an amino acid-based formula supplemented with two human milk oligosaccharides on growth, tolerability, safety, and gut microbiome in infants with cow’s milk protein allergy. Nutrients.

[28] Román E., Moreno Villares J.M., Domínguez Ortega F., Carmona Martínez A., Picó Sirvent L., Santana Sandoval L. (2020). Real-world study in infants fed an infant formula with two human milk oligosaccharides. Nutr. Hosp..

[29] Nowak-Wegrzyn A., Czerkies L., Reyes K., Collins B., Heine R.G. (2019). Confirmed hypoallergenicity of a novel whey-based extensively hydrolyzed infant formula containing two human milk oligosaccharides. Nutrients.

[30] Storm H.M., Shepard J., Czerkies L.M., Kineman B., Cohen S.S., Reichert H. (2019). 2′-fucosyllactose is well tolerated in a 100% whey, partially hydrolyzed infant formula with Bifidobacterium lactis: a randomized controlled trial. Glob. Pediatr. Health.

[31] Prieto A.P. (2005). In vitro and clinical experiences with a human milk oligosaccharide, lacto-N neoTetraose, and fructooligosaccharides. Foods Food Ingred. J. Jpn..

[32] Kajzer J., Oliver J., Marriage B. (2016). Gastrointestinal tolerance of formula supplemented with oligosaccharides. FASEB J..

[33] Ramirez-Farias C., Oliver J.S., Schlezinger J., Stutts J.T. (2024). Tolerance of infants fed a hydrolyzed rice infant formula with 2′-fucosyllactose (2′-FL) human milk oligosaccharide (HMO). Nutrients.

[34] Jochum F., Meyer-Krott M., Hübler T., Lorenz M., Bedikian R., Zakarian J. (2023). Real-world evidence study on tolerance and growth in infants fed an infant formula with two human milk oligosaccharides vs mixed fed and exclusively breastfed infants. Mol. Cell Pediatr..

[35] Giorgetti A., Paganini D., Nyilima S., Kottler R., Frick M., Karanja S. (2023). The effects of 2′-fucosyllactose and lacto-N-neotetraose, galacto-oligosaccharides, and maternal human milk oligosaccharide profile on iron absorption in Kenyan infants. Am. J. Clin. Nutr..

[36] Scheuchzer P., Sinawat S., Donzé A.S., Zeder C., Sabatier M., Garcia-Garcera M. (2024). Iron absorption from an iron-fortified follow-up formula with and without the addition of a synbiotic or a human-identical milk oligosaccharide: a randomized crossover stable isotope study in Young Thai children. J. Nutr..

[37] Marriage B.J., Buck R.H., Goehring K.C., Oliver J.S., Williams J.A. (2015). Infants fed a lower calorie formula with 2′FL show growth and 2′FL uptake like breast-fed infants. J. Pediatr. Gastroenterol. Nutr..

[38] Goehring K.C., Marriage B.J., Oliver J.S., Wilder J.A., Barrett E.G., Buck R.H. (2016). Similar to those who are breastfed, infants fed a formula containing 2′-fucosyllactose have lower inflammatory cytokines in a randomized controlled trial. J. Nutr..

[39] Hill D.R., Buck R.H. (2023). Infants fed breastmilk or 2′-FL supplemented formula have similar systemic levels of microbiota-derived secondary bile acids. Nutrients.

[40] Puccio G., Alliet P., Cajozzo C., Janssens E., Corsello G., Sprenger N. (2017). Effects of infant formula with human milk oligosaccharides on growth and morbidity: A randomized multicenter trial. J. Pediatr. Gastroenterol. Nutr..

[41] Berger B., Porta N., Foata F., Grathwohl D., Delley M., Moine D. (2020). Linking human milk oligosaccharides, infant fecal community types, and later risk to require antibiotics. mBio.

[42] Dogra S.K., Martin F.P., Donnicola D., Julita M., Berger B., Sprenger N. (2021). Human milk oligosaccharide-stimulated Bifidobacterium species contribute to prevent later respiratory tract infections. Microorganisms.

[43] Martin F.P., Tytgat H.L., Krogh Pedersen H., Moine D., Eklund A.C., Berger B. (2022). hosts. Host-microbial co-metabolites modulated by human milk oligosaccharides relate to reduced risk of respiratory tract infections. Front Nutr.

[44] Vandenplas Y., de Halleux V., Arciszewska M., Lach P., Pokhylko V., Klymenko V. (2020). A partly fermented infant formula with postbiotics including 3′-GL, specific oligosaccharides, 2′-FL, and milk fat supports adequate growth, is safe and well-tolerated in healthy term infants: a double-blind, randomised, controlled, multi-country trial. Nutrients.

[45] Wallingford J.C., Neve Myers P., Barber C.M. (2022). Effects of addition of 2-fucosyllactose to infant formula on growth and specific pathways of utilization by Bifidobacterium in healthy term infants. Front Nutr.

[46] Alliet P., Vandenplas Y., Roggero P., Jespers S.N., Peeters S., Stalens J.P. (2022). Safety and efficacy of a probiotic-containing infant formula supplemented with 2′-fucosyllactose: a double-blind randomized controlled trial. Nutr. J..

[47] Parschat K., Melsaether C., Jäpelt K.R., Jennewein S. (2021). Clinical evaluation of 16-week supplementation with 5HMO-Mix in healthy-term human infants to determine tolerability, safety, and effect on growth. Nutrients.

[48] Lasekan J., Choe Y., Dvoretskiy S., Devitt A., Zhang S., Mackey A. (2022). Growth and gastrointestinal tolerance in healthy term infants fed milk-based infant formula supplemented with five human milk oligosaccharides (HMOs): a randomized multicenter trial. Nutrients.

[49] Bosheva M., Tokodi I., Krasnow A., Pedersen H.K., Lukjancenko O., Eklund A.C. (2022). Infant formula with a specific blend of five human milk oligosaccharides drives the gut microbiota development and improves gut maturation markers: a randomized controlled trial. Front Nutr.

[50] Vandenplas Y., Żołnowska M., Berni Canani R., Ludman S., Tengelyi Z., Moreno-Álvarez A. (2022). Effects of an extensively hydrolyzed formula supplemented with two human milk oligosaccharides on growth, tolerability, safety and infection risk in infants with cow’s milk protein allergy: a randomized, multi-center trial. Nutrients.

[51] Boulangé C.L., Pedersen H.K., Martin F.P., Siegwald L., Pallejà Caro A., Eklund A.C. (2023). An extensively hydrolyzed formula supplemented with two human milk oligosaccharides modifies the fecal microbiome and metabolome in infants with cow’s milk protein allergy. Int. J. Mol. Sci..

[52] Hascoët J.M., Chevallier M., Gire C., Brat R., Rozé J.C., Norbert K. (2022). Use of a liquid supplement containing 2 human milk oligosaccharides: the first double-blind, randomized, controlled trial in pre-term infants. Front Pediatr.

[53] Nuzhat S., Hasan S.M., Palit P., Islam M.R., Mahfuz M., Islam M.M. (2023). Effects of probiotic and synbiotic supplementation on ponderal and linear growth in severely malnourished young infants in a randomized clinical trial. Sci. Rep..

[54] Leung T.F., Ulfman L.H., Chong M.K., Hon K.L., Khouw I.M., Chan P.K. (2020). A randomized controlled trial of different young child formulas on upper respiratory and gastrointestinal tract infections in Chinese toddlers. Pediatr Allergy Immunol.

[55] Holst AQ, Myers P, Rodríguez-García P, Hermes GDA, Melsaether C, Baker A, Jensen SR, Parschat K (2023). Infant formula supplemented with five human milk oligosaccharides shifts the fecal microbiome of formula-fed infants closer to that of breastfed infants. Nutrients.

[56] Salminen S., Collado M.C., Endo A., Hill C., Lebeer S., Quigley E.M. (2021). The International Scientific Association of Probiotics and Prebiotics (ISAPP) consensus statement on the definition and scope of postbiotics. Nat. Rev. Gastroenterol. Hepatol..

[57] Subramanian S., Huq S., Yatsunenko T., Haque R., Mahfuz M., Alam M.A. (2014). Persistent gut microbiota immaturity in malnourished Bangladeshi children. Nature.

[58] Prentice P.M., Schoemaker M.H., Vervoort J., Hettinga K., Lambers T.T., van Tol E.A. (2019). Human milk short-chain fatty acid composition is associated with adiposity outcomes in infants. J. Nutr..

[59] Bachem A., Makhlouf C., Binger K.J., de Souza D.P., Tull D., Hochheiser K. (2019). Microbiota-derived short-chain fatty acids promote the memory potential of antigen-activated CD8^+^ T cells. Immunity.

