# Supplementary material for: Limited Evidence of Benefits from Clinical Trials of Human-Identical Milk Oligosaccharides for Infants
Source: Adv Nutr. 2026 Jan 14;17(3):100593. doi: 10.1016/j.advnut.2026.100593 (PMC12934283; doi:10.1016/j.advnut.2026.100593)
Supplement: multimedia component 1 [file mmc1.docx]

#### **Manuscript Title: Limited evidence of benefits from clinical trials of human-identical milk oligosaccharides for infants**

**First author**: Rupak Shivakoti

#### **Supplementary Table 1. Search terms used to search clinical trials and meta-analysis**

| **COMBINED FINAL SEARCH STRATEGY** |
| --- |
| **Search terms:** |
| ("human milk oligosaccharide*" OR "2 fucosyllactose"OR "2 fl" OR "3-fucosyllactose"[All Fields] OR "3-FL"[All Fields] OR "3 galactosyllactose"[All Fields] OR "3 gl"[All Fields] OR "3 sialyllactose"[All Fields] OR "3 sl"[All Fields] OR "6 sialyllactose"[All Fields] OR "6 sl"[All Fields] OR "difucosyllactose"[All Fields] OR "DFL"[All Fields] OR "lacto-N-tetraose"[All Fields] OR "LNT"[All Fields] OR "lacto-N-neotetraose"[All Fields] OR "LNnT"[All Fields] OR (("milk, human"[MeSH Terms] OR ("milk"[All Fields] AND "human"[All Fields]) OR "human milk"[All Fields] OR ("human"[All Fields] AND "milk"[All Fields])) AND ("oligosaccharides"[MeSH Terms] OR "oligosaccharides"[All Fields] OR "oligosaccharide"[All Fields] OR "oligosaccharidic"[All Fields])) OR ("2 fucosyllactose"[Supplementary Concept] OR "2 fucosyllactose"[All Fields] OR "2 fucosyllactose"[All Fields]) OR "2 fl"[All Fields] OR ("3-fucosyllactose"[Supplementary Concept] OR "3-fucosyllactose"[All Fields] OR "3-fucosyllactose"[All Fields]) OR "3-FL"[All Fields] OR ("3 galactosyllactose"[Supplementary Concept] OR "3 galactosyllactose"[All Fields] OR "3 galactosyllactose"[All Fields]) OR "3 gl"[All Fields] OR ("3 sialyllactose"[Supplementary Concept] OR "3 sialyllactose"[All Fields] OR "3 sialyllactose"[All Fields]) OR "3 sl"[All Fields] OR ("6 sialyllactose"[Supplementary Concept] OR "6 sialyllactose"[All Fields] OR "6 sialyllactose"[All Fields]) OR "6 sl"[All Fields] OR "difucosyllactose"[All Fields] OR "DFL"[All Fields] OR ("lacto-N-neotetraose"[Supplementary Concept] OR "lacto-N-neotetraose"[All Fields] OR "lacto-N-tetraose"[All Fields]) OR "LNT"[All Fields] OR ("lacto-N-neotetraose"[Supplementary Concept] OR "lacto-N-neotetraose"[All Fields] OR "lacto-N-neotetraose"[All Fields]) OR ("lacto-N-neotetraose"[Supplementary Concept] OR "lacto-N-neotetraose"[All Fields] OR "LNnT"[All Fields])) |
| **Filters applied:** |
| - Study type: Clinical Trial, Meta-Analysis, Randomized Controlled Trial - Language: English - Species: Humans - Ages: Newborn: birth-1 month, Infant: birth-23 months, Infant: 1-23 months, Preschool Child: 2-5 years. |

**Supplementary 1 Legend**: The search terms used is detailed above, along with the filters applied related to study type, language of the study, focus on humans and age group.
